# Production of Pectate Lyase by *Penicillium viridicatum RFC3* in Solid-State and Submerged Fermentation

**DOI:** 10.1155/2010/276590

**Published:** 2010-06-29

**Authors:** Viviani Ferreira, Roberto da Silva, Dênis Silva, Eleni Gomes

**Affiliations:** Laboratory of Biochemistry and Applied Microbiology, Ibilce, São Paulo State University-Unesp, Rua Cristovao Colombo, 2265, Jd. Nazareth, 15054-000 São José do Rio Preto, SP, Brazil

## Abstract

Pectate lyase (PL) was produced by the filamentous fungus *Penicillium viridicatum* RFC3 in solid-state cultures of a mixture of orange bagasse and wheat bran (1 : 1 w/w), or orange bagasse, wheat bran and sugarcane bagasse (1 : 1 : 0.5 w/w), and in a submerged liquid culture with orange bagasse and wheat bran (3%) as the carbon source. PL production was highest (1,500 U  mL^−1^ or 300 Ug^−1^ of substrate) in solid-state fermentation (SSF) on wheat bran and orange bagasse at 96 hours. PL production in submerged fermentation (SmF) was influenced by the initial pH of the medium. With the initial pH adjusted to 4.5, 5.0, and 5.5, the peak activity was observed after 72, 48, and 24 hours of fermentation, respectively, when the pH of the medium reached the value 5.0. PL from SSF and SmF were loaded on Sephadex-G75 columns and six activity peaks were obtained from crude enzyme from SSF and designated PL I, II, III, IV, V, and VI, while five peaks were obtained from crude enzyme from SmF and labeled PL  I′, II′, III′, IV′, and VII′. Crude enzyme and fraction III from each fermentative process were tested further. The optimum pH for crude PL from either process was 5.5, while that for PL III was 8.0. The maximum activity of enzymes from SSF was observed at 35°C, but crude enzyme was more thermotolerant than PL III, maintaining its maximum activity up to 45°C. Crude enzyme from SmF and PL III′ showed thermophilic profiles of activity, with maximum activity at 60 and 55°C, respectively. In the absence of substrate, the crude enzyme from SSF was stable over the pH range 3.0–10.0 and PL III was most stable in the pH range 4.0–7.0. Crude enzyme from SmF retained 70%–80% of its maximum activity in the acid-neutral pH range (4.0–7.0), but PIII showed high stability at alkaline pH (7.5–9.5). PL from SSF was more thermolabile than that from SmF. The latter maintained 60% of its initial activity after 1 h at 55°C. The differing behavior of the enzymes with respect to pH and temperature suggests that they are different isozymes.

## 1. Introduction

The pectinolytic enzyme group includes protopectinases that degrade insoluble pectin, esterases that catalyse the de-esterification of pectin by removing the methoxyl esters groups and depolymerases that cleave *α*- (1 → 4-) glycosidic bonds by hydrolysis and transelimination mechanisms. The lyases break down the glycosidic bonds of the pectates or pectins at C-4 and eliminate H from C-5, releasing a 4,5-unsaturated product [1]. These enzymes belong to various classes: pectate disaccharide lyase (exopectate lyase—ExoPGL) E.C. 4.2.2.9, pectin lyase (Endo-pectin lyase—EndoPMGL) E.C. 4.2.2.10, and pectate lyase (Endogalacturonate transeliminase—EndoPGL) E.C. 4.2.2.2 (Enzyme Nomenclature database of IUBMB). 

Solid-state fermentation (SSF) is a process that involves solid matrix and occurs in the absence or near absence of any fluid in the space between substrate particles. In this system, water is present in the solid substrate, whose capacity for liquid retention varies with the type of material. In contrast, in submerged fermentation (SmF), the nutrients and microorganisms are both submerged in water [2].

There has been much discussion of the advantages and disadvantages of using one or the other fermentative process [3]. It has been argued that SSF increases the amount of enzyme obtained, relative to SmF [4, 5]. Some investigations have shown that enzyme production is more sensitive to catabolite repression in SmF [6, 7]. Intrinsic properties of microbial extracellular protein molecules, for example, temperature and pH optima for activity, thermostability, stability in various pH ranges, and substrate affinity are also influenced by the type of fermentative process used for their production [8, 9]. Besides, Aguilar et al. [4] reported that the expression of proteins may differ in SSF and SmF.

Waste material from agroindustrial processing may be used as the substrate for microbial growth in SSF or SmF. The organic matter in this material is used as a source both of energy for growth and of carbon and nutrient for synthesis of cell biomass and other products of microbial metabolism, so that the waste is upgraded and valuable products may be synthesized [10, 11]. 

This paper reports the production and physicochemical properties of pectate lyase (PL) obtained from *P. viridicatum *RFC3 by solid-state fermentation and submerged fermentation on a mixture of wheat bran and solid waste (*bagasse*) from oranges as the carbon source.

## 2. Material and Methods

### 2.1. Microorganism

The microorganism used was *Penicillium viridicatum* RFC3, a strain isolated from decaying vegetable in São José do Rio Preto, SP, Brazil, and maintained as a stock culture on Potato Dextrose Agar (PDA-Oxoid) containing 0.3% citrus pectin.

### 2.2. Fermentations for Enzyme Production

 The medium composition and processing conditions for solid-state fermentation (SSF) were described previously [12]. 5 g of a mixture (1 : 1 w/w) of wheat bran and orange bagasse (pulp and rind from juiced oranges) or of (1 : 1 : 0.5 w/w) wheat bran, orange bagasse and sugarcane bagasse, were placed in 250 mL Erlenmeyer flasks, sterilized at 120°C for 20 minutes and then hydrated to 70% moisture with a sterile solution of (g/L) 10 (NH_4_)_2_SO_4_, 10 MgSO_4_ · 7H_2_O, and 10 NH_4_H_2_PO_4_ (pH 4.6). The substrate was inoculated with a suspension of conidia in 0.1% Tween 80, equivalent to 10^7^ spores per gram dry substrate, and cultured at 28°C for 14 days. Every two days, one flask was removed and the fermented material therein was mixed with 8 mL distilled water per gram of substrate, stirred for 30 minutes, filtered and centrifuged at 10,000 × g for 15 minutes at 4°C. The supernatant was used as the crude enzyme solution from which the final pH of the medium and its reducing sugar content were determined.

The submerged fermentation (SmF) was carried out in 125 mL Erlenmeyer flasks, each with 20 mL of medium containing (g/L) 10 (NH_4_)_2_SO_4_, 10 MgSO_4_ · 7H_2_O, 10 NH_4_H_2_PO_4_, 15 orange bagasse, and 15 wheat bran. The last two were first ground and the particles sieved through a Bender USS 230 strainer. The pH was corrected to 4.5, 5.0, or 5.5 in the unbuffered media by use of HCl and NaOH. The media were inoculated with a suspension of conidia in 0.1% Tween 80, equivalent to 10^7^ spores per mL of medium. Fermentation was carried out in a rotary shaker at 100 rpm for 120 hours at 28°C. Every 24 hours, one flask was removed and the biomass was separated by filtering through Whatman No. 1 paper in a Büchner funnel. The filtrate was used to evaluate enzyme activity and to determine the final pH and reducing sugar content.

The fermentation experiments were performed in triplicate and the results are reported as means.

### 2.3. Enzyme Activity Measurements

Pectate lyase (PL) was assayed by measuring the increase in absorbance at 235 nm of a solution of substrate (0.8 mL 1% citric pectin [Sigma] in 0.2 M Tris/HCl buffer, pH 7.5) hydrolyzed by 0.2 mL enzyme solution, at 50°C. One unit of enzymatic activity (U) was defined as the amount releasing 1 *μ*mol of unsaturated uronide per minute, based on the molar extinction coefficient (5500) of the unsaturated products [13]. 

Enzyme production was expressed in units per milliliter of crude enzyme solution (U/mL) and in units per gram of substrate (U/g).

### 2.4. Enzyme Fraction Separation

The crude enzyme solutions obtained from SSF (medium composed of orange bagasse and wheat bran, after 336 hours of fermentation) and SmF (pH 4.5, 72 hours of fermentation) were concentrated by ultrafiltration in a QuixStand Benchtop membrane system (GE Healthcare) with a 10 kDa cut-off. The concentrate was dialyzed against 10 mM acetate buffer (pH 5.0) for 24 hours and then loaded on a Sephadex G-75 (Pharmacia) column (2.6 × 90 cm) equilibrated with 20 mM acetate buffer (pH 5.0) and eluted with the same buffer at a flow rate of 0.3 mL/minute. Fractions of 4 mL were collected and assayed for PL activity, as described above. Protein elution was checked by protein determination. The object of this procedure was to estimate the number of isoforms of PL present in the crude enzyme solution.

### 2.5. Enzyme Characterization

PL activity was assayed at pH values ranging from 3 to 11, in 200 mM acetate buffer (pH 3.0–5.5), citrate buffer (pH 6.0–7.0), Tris-HCl (pH 7.5–9.0), and glycine-NaOH (pH 9.5–11.0), at 40°C, with 87%-esterified (D.E.) citric pectin (Sigma) as substrate. The temperature effect on PL activity was determined in the acetate buffer, incubated at temperatures from 30°C to 75°C, at the pH optimum (5.0 for crude enzyme and 8.0 for PLIII). Both assays were carried out as described above. 

The thermal stability was estimated by measuring the residual activity of the enzyme after it had been held at temperatures between 10°C and 80°C for 1 hour, in the absence of substrate. Residual PL activity was determined at optimum pH and temperature, using 26% D.E. citrus pectin as substrate. 

Variation of enzyme stability with pH was evaluated by mixing (1 : 1 v/v) enzyme solution with 0.1 M buffer solutions at pH 3.0–5.0 (sodium acetate), pH 5.0–7.0 (citrate-phosphate), pH 7.0–8.5 (Tris-HCl), and pH 8.5–11.0 (glycine-NaOH) and maintaining these solutions at 25°C for 24 hours. An aliquot of 0.1 mL was taken to determine the remaining activity at the optimum pH and temperature.

The effects of various metal ions on enzyme activity were evaluated at a concentration of 2 mM in the reaction mixture, with FeCl_3_ · 6H_2_O, Ag_2_SO_4_, CaSO_4_ · 2H_2_O, MgSO_4_ · H_2_O, MnSO_4_ · H_2_O, ZnSO_4_ · 7H_2_O, K_2_SO_4_, HgSO_4_, CaCl_2_, and EDTA [12].

The products of the hydrolysis of 26% esterified citrus pectin by PL were analyzed by paper chromatography on Whatman No. 1 paper, with ethyl acetate/isopropanol/water (6 : 3 : 1, by volume) as the mobile phase [14].

The substrate specificity was evaluated using polygalacturonic acid and citrus pectin (Sigma) with 26% and 92% D.E. as substrates under optimal conditions for enzyme activity.

### 2.6. Analytical Methods

The growth of the culture was evaluated in terms of dry weight. Fermented material from SmF was filtered through Whatman No. 1 filter paper and the biomass was washed with chilled deionized water, vacuum filtered and dried at 65°C to a constant weight.

Reducing sugar was measured by the dinitrosalicylic acid method [15] and protein by the Bradford [16] method.

## 3. Results and Discussion

### 3.1. Enzyme Production by SSF and SmF

**Figure 1:**
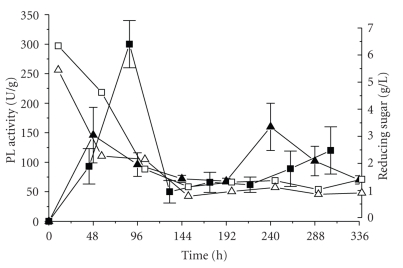
PL production by *P. viridicatum* RFC3 in solid state fermentation (SSF), using mixtures (w/w) of wheat bran and orange bagasse 1 : 1 (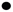 PL activity; 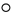 reducing sugar) or wheat bran, orange bagasse and sugarcane bagasse 1 : 1 : 0.5 (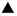 = PL activity; 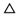 = reducing sugar).

The results from the solid-state fermentation are shown in Figure 1(a). PL production on wheat bran and orange bagasse mixture peaked sharply at 96 hours, but when wheat bran, orange bagasse, and sugarcane bagasse mixture were used, two peaks were observed, after 48 and at 240 hours of growth. The maximum activity was 1,500 U/mL (or 300 U/g of substrate), obtained in the first medium. The initial free reducing sugar content of the fermentation medium was 5.5 g/L for the mixture of orange bagasse, wheat bran, and sugar cane bagasse, falling to 2.1 g/L after 48 hours of fermentation, and 6.2 g/L for the mixture of orange bagasse and wheat bran, falling to 1.5 g/L after 96 hours (Figure 1(b)). Since the maximal activities were observed, respectively, after 48 and 96 hours, when the free reducing sugar content was low, the expression of lyase appears to be inversely with readily available carbons.

**Figure 2:**
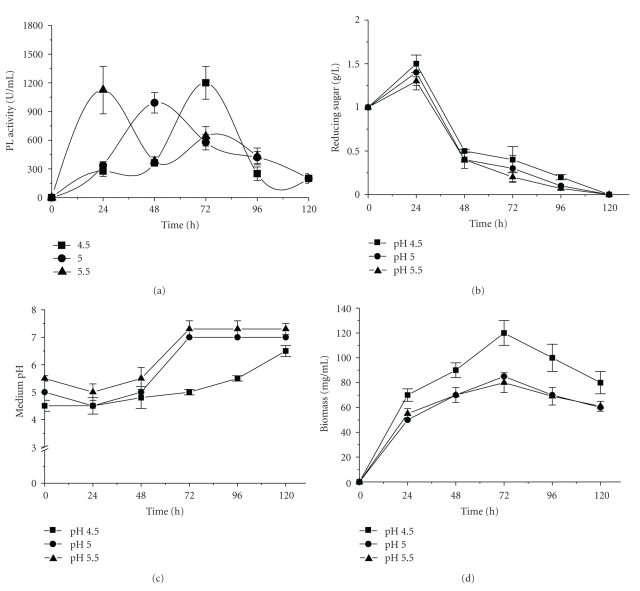
PL production by *P. viridicatum* RFC3 in submerged fermentation (SmF), using wheat bran and orange bagasse (1 : 1) as carbon source. (a) PL activity; (b) reducing sugar; (c) medium pH; and (d) biomass production. (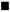) = pH 4.5;( 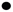) = pH 5.0; and (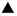) = pH 5.5.

It is seen in Figure 2 that the profile of PL production through SmF of course was influenced by the initial pH of the medium, though the maximum activity (1000–1200 U/mL) did not vary much. The peak activities obtained when the initial pH was 4.5, 5.0, and 5.5 were observed after 72, 48, and 24 hours of fermentation, respectively (Figure 2(a)). The reducing sugar concentration in the medium during the fermentation showed the same profile for all initial pH values (Figure 2(b)). On the other hand, when these data are compared with those in Figure 2(c), it is observed that the maximum PL activity occurred in all three media when the medium pH reached 5.0 (Figure 2(c)). Other authors have described pH 5.0 as the best for fungal polygalacturonase production [17–19], whereas pectin lyase production was highest at neutral-alkaline pH [20–22]. 

The growth of the microorganism was higher at pH 4.5 after 72 hours of culture and was not different between growth at pH 5.0 and 5.5 but profiles of enzyme production were significantly affected by the pH of the medium since the peak of enzyme activity at pH 5.5 was obtained after 24 hours, at pH 5.0 after 48 hours and at pH 4.5, 72 hours. It is important to observe that in this time the pH of the medium was around 5.0 (Figure 2(d)). The observed effect of pH must relate to mechanisms regulating enzyme synthesis and/or secretion. Regulation of fungal enzyme gene expression by ambient pH has been reported for glucoamylase in *A. niger *[23], alkaline and acid phosphatases in *A. nidulans* [24] ), and xylanases in *Trichoderma reesei* [25]. With respect to pectinases, it has been reported that ambient pH might activate genes encoding various endo and exopolygalacturonases in phytopathogenic fungi such as *Alternaria alternate*, *A. citri* [26], *Sclerotinia sclerotium* [27], *Colletotrichum* sp [28], and *Botrytis cinerea* [29]. It has also been demonstrated that the expression of certain specific isoforms of polygalacturonases is highly favored in acidic media and that the environmental pH is lowered by oxalic acid production [27], while other isoforms are expressed after the ambient pH has been raised [26]. The other studies will be necessary to confirm this regulation model for PL from *P. viridicatum*. 

In light of the present results, one can conclude that PL production by *P. viridicatum* in SmF is apparently unaffected by reducing sugar concentration, mycelial growth, or fermentation time, but is controlled by the pH of the medium.

### 3.2. Separation of PL Fractions

**Figure 3:**
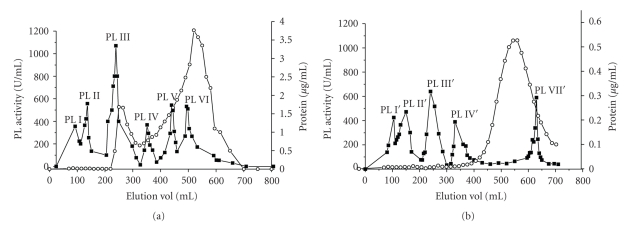
Elution of PL activity from Sephadex G75 chromatography columns (3.0 × 80.0 cm) previously equilibrated with 20 mM acetate buffer, pH 5.0. (a) PL from SSF; (b) PL from SmF. (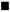) = Pl activity; (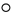) = Protein.

In order to determine the number of PL isoforms present in the culture of *P. viridicatum*, the crude enzymes from SSF and SmF were loaded on Sephadex-G75 columns. Six activity peaks were obtained from the crude enzyme produced by SSF with wheat bran and orange bagasse mixture as substrate, after 96 hours of fermentation. They are labeled PL I, II, III, IV, V, and VI (Figure 3(a)). Five peaks were obtained by the same procedure from crude enzyme produced by 96 hours of submerged fermentation and were designated PL  I′, II′, III′, IV′, and VII′ (Figure 3(b)). 

Production of several isoforms of the same enzyme occurs in many species of fungi and bacteria, and they often differ in their pI, molecular weight, and substrate affinity. This variation in the isoforms of extracellular enzymes can be attributed to many factors, such as the presence of several genes, different degrees of glycosylation, posttranscriptional processing, or proteolysis after secretion [14, 30]. Multiple isoforms of secreted pectinases are usually obtained in fungal culture media [11, 22, 31, 32] since the infection of host living tissue by a pathogen and colonization of plant cells decaying by saprophytic microorganisms is facilitated if a sufficient quantity and a number of isoform types of pectinase are produced [33]. 

The presence of several PLs in the culture medium of *P. viridicatum *is consistent with other *Penicillium* species such as *P. adametzii, P. citrinun*, *P*. *janthinellum* [34], and *P. griseoroseum* [35], which are saprophytes or associated with living tissue. 

Comparing the positions of eluted fractions (Figure 3), the peaks I′, II′, III′, and IV′ (SmF) are equivalent to those observed in the chromatogram of the SSF product, but PL VII′ was detected only in the enzyme from SmF while the peaks V and VI were observed only in the chromatogram of the SSF enzyme. 

One of the main characteristics of SSF, the low water potential (*a*_*w*_) of the solid medium, influences physiological aspects of the microorganism such as metabolite and enzyme production and extracellular enzyme activity [3]. Some proteins can be expressed during SSF incubation but not in submerged cultures [4] and molecular studies have demonstrated that conditions of low *a*_*w*_ can induce specific gene expression [36, 37]. It has been reported that the production of endopolygalacturonase, exopolygalacturonase, and pectin lyase by *A. niger* was higher in SSF than in SmF [4, 5].

### 3.3. Comparison of Some Properties of Crude Enzyme and Fraction III of PLs from SSF and SmF

**Figure 4:**
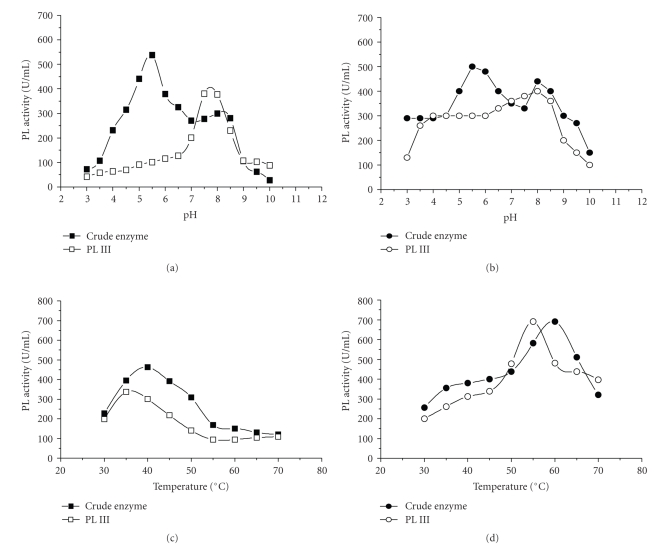
Physicochemical activity profiles of PLs obtained from *P. viridicatum* by SSF (a; c) and SmF (b; d). ( 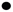/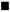) = crude enzyme; (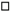/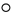) = fraction III obtained by Sephadex-G75 chromatography.

The optimum pH for activity of crude PL from either fermentative process was 5.5 and that for PL III and III′ was 8.0, although PL III′ from SmF exhibited an unusual pH profile, with 50% of maximum activity in the pH range 3.0–6.0 (Figures 4(a) and 4(b)).

The maximum activity of both enzymes from SSF was observed at 35°C, although crude enzyme was more thermo-tolerant than PL III, maintaining its maximum activity up to 45°C (Figure 4(c)). However, crude enzyme and PL III′, from SmF, had thermophílic profiles of activity with maximum activity at 60 and 55°C, respectively (Figure 4(d)). 

**Figure 5:**
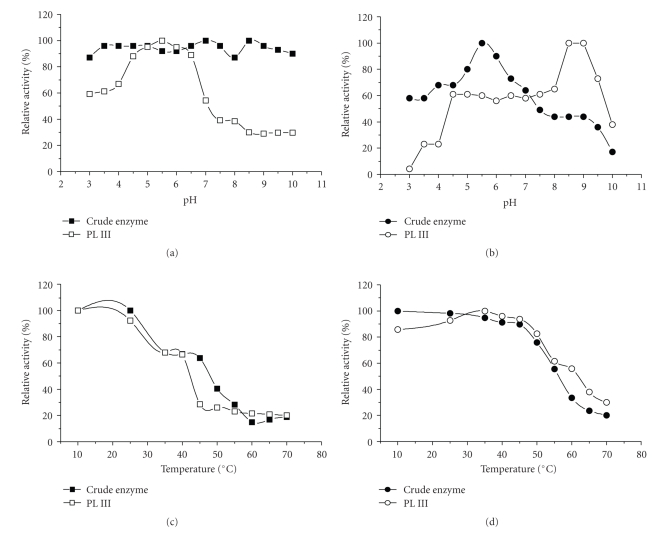
Physicochemical profiles of stability of PLs obtained from *P. viridicatum* by SSF (a; c) and SmF (b; d). (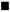/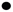) = crude enzyme; (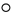/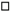) = fraction III obtained by Sephadex-G75 chromatography.

In the absence of substrate, the crude enzyme from SSF was stable from pH 3.0 to 10.0 and PL III showed maximum stability in the acid-neutral pH range (Figure 5(a)). Crude enzyme from SmF retained 70%–80% of maximum activity in the acid-neutral pH range (4.0 to 7.0) but PL III′ showed high stability at alkaline pH (7.5–9.5) (Figure 5(b)). PL from SSF was more thermolabile than that from SmF. The latter maintained 60% of its initial activity after 1 hour at 55°C (Figures 5(c) and 5(d)). 

Pectin lyases produced by filamentous fungi of several types have optimum pH between 6.0 (*P. italicum*) and 11 (*Thermoascus aurantiacus*) The optimal temperature for activity is normally between 40 and 50°C for mesophilic strains and 65°C for thermophilic fungi such as *T. aurantiacus* [14, 20], although PL from the mesophilic *Fusarium moniliforme* shows maximum activity at 70°C [38]. According to a review by Jayani et al. [39], fungal PLs are stable from acidic (4.0) to alkaline (8.0) pH, but their activity and stability may peak in different pH ranges. For example, *T. aurantiacus* produces PL with optimum activity at pH 10.5–11 and highest stability at pH 4.0.

The effects of a variety of cations and EDTA on the activity of PL III from SSF and III′ from SmF were tested. The resulting activities relative to normal controls are given in Table 1. The ions Hg^2+^, Mg^2+^, Mn^2+^, Ag^+^, and Fe^3+^ inhibited the enzyme activity by around 80%–97%. It has long been known that some metal ions are inhibitors of a large range of enzymes, including pectinase [40]. PL III′ activity (SmF) was enhanced 17% by Ca^2+^ and 84% by K^+^, but that of PIII (SSF) was reduced 95.4% by Ca^2+^ and 50% by K^+^. 

**Figure 6:**
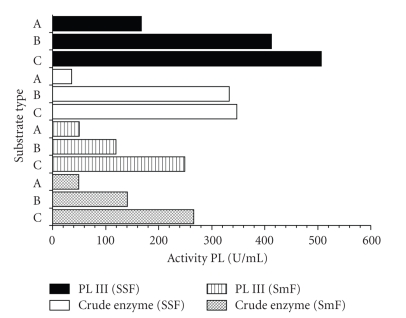
Substrate specificity of crude enzyme and P LIII produced by *P. viridicatum* RFC3 in solid-state (SSF) and submerged fermentation (SmF): (A) = 92%-esterified citrus pectin (Sigma); (B) = 26%-esterified citrus pectin (Sigma); and (C) = Polygalacturonic acid.

Data shown in Figure 6 suggest that there was no difference in substrate preference among the crude and fractionated PLs from SSF and SmF. The higher activity on polygalacturonic acid than on high-D.E. pectin indicated that these enzymes are pectate lyases.

Even though PL III from SSF and PL III′ from SmF were eluted at the same volume from the chromatography column and seemingly have the same molecular weight and substrate specificity, the differences in behavior of the two enzymes with respect to pH, temperature, and metal ion effects suggest that they are different isozymes.

## Figures and Tables

**Table 1:** Influence of ions on the activity of fraction III obtained by Sephadex-G75 chromatography of PLs produced by SSF and SmF.

Ions	PL III′ (SmF)	PL III (SSF)
Control	100	100
Fe^3+^	2.9	14.3
Ag^+^	8.7	3.1
K^+^	184.0	50.0
Mg^2+^	14.9	6.3
Mn^2+^	24.9	7.4
Zn^2+^	18.7	5.1
Hg^2+^	19.5	5.0
Ca^2+^	117.1	4.6
EDTA	14.0	36.5
